# Prognostic factors for outcome after septoplasty in 888 patients from the Swedish National Septoplasty Register

**DOI:** 10.1007/s00405-019-05440-6

**Published:** 2019-04-29

**Authors:** Lars Pedersen, L. Schiöler, S. Finjan, Å. Davidsson, O. Sunnergren, K. Holmberg, C. Ahlström Emanuelsson, J. Hellgren

**Affiliations:** 1 Department of Otorhinolaryngology, Head and Neck Surgery, Institute of Clinical Sciences, Sahlgrenska Academy, University of Gothenburg, Gröna Stråket 9, 413 45 Göteborg, Sweden; 2 Department of Occupational and Environmental Medicine, Sahlgrenska Academy, University of Gothenburg, Göteborg, Sweden; 3 School of Medical Sciences, Örebro University, Campus USÖ, Örebro, Sweden; 4 Department of Otorhinolaryngology, Ryhov County Hospital, Jönköping, Sweden; 5 Department of Otorhinolaryngology, Head and Neck Surgery, Skane University Hospital, Lund University, Lund, Sweden

**Keywords:** Nasal breathing, Nasal obstruction, Outcome, Register study, Septoplasty

## Abstract

**Background:**

The aim of this study was to identify predictors of outcome after septoplasty in 888 patients from the Swedish National Septoplasty Register.

**Methodology:**

This is an observational register study analysing data from patients undergoing septoplasty in Sweden between 2015 and 2016. The patients reported severity of nasal obstruction (mild, moderate, severe) pre- and again 12 months postoperatively (none, mild, moderate, severe), unplanned visits within 30 days after surgery. The examining doctor reported co-morbidities such as allergic rhinitis and snoring. The primary end-point was one level improvement of the nasal obstruction 12 months after surgery.

**Results:**

Nasal obstruction had improved in 63% 12 months after surgery. Twelve months after surgery, 81% with severe nasal obstruction and 31% with mild nasal obstruction before surgery had improved. Only 56% reported that the results of the surgery were as they had expected. Higher patient age at surgery, no unplanned visits within 1 month of surgery and activity limitation before surgery were associated with improvements in nasal breathing in the logistic regression model.

**Conclusion:**

Septoplasty should be offered to patients with severe nasal obstruction and surgery should be avoided in mild nasal obstruction confirmed by both an improvement in nasal obstruction and patient expectations in this study.

## Introduction

Septoplasty is the main surgical procedure to relieve structural nasal obstruction due to a nasal septum deviation. In some cases, additional turbinate surgery is performed. Numerous studies from different health-care systems have shown that around 2/3 of patients undergoing septoplasty with or without turbinate surgery experience an improvement in nasal breathing in short- and long-term follow-up studies [1–5]. Assessments with objective parameters such as rhinomanometry before and after surgery show varying results [4, 6, 7]. Some factors associated with a poorer perception of the surgical result, such as younger patient age and making unplanned visits to health care within 1 month of surgery, have been identified [1]. The extent to which the patient-rated severity of the nasal obstruction preoperatively predicts the postoperative result has not previously been studied extensively in large patient cohorts. As septoplasty is a common procedure, it is performed by a large number of ENT surgeons with a varying degree of experience, from doctors in training to experienced nasal surgeons. The ability to identify general predictors and associated factors of the surgical outcome beyond individual surgical skill is, therefore, important when selecting patients for septoplasty.

The National Swedish Septoplasty Register (SNSR) was started by the Swedish Association for Otorhinolaryngology, Head and Neck Surgery (SFOHH) in 1997. The SNSR is monitored by an expert group of Swedish rhinologists and funded by the Swedish Association of Local Authorities and Regions. Septoplasties performed in public hospitals and in private practice in Sweden are reported to the register. Since 2013, the SNSR has included questions regarding the severity of nasal obstruction pre- and postoperatively, co-morbidities and questions regarding the impact of nasal obstruction on daily activities and sleep. The upgraded SNSR enables extended analyses of parameters associated with the surgical result after septoplasty in a prospective design. The main aim of the present study was to identify general predictors and associated factors of the surgical result in a large national sample of patients undergoing septoplasty in Sweden, with the emphasis on self-reported severity of nasal obstruction pre- and 12 months postoperatively. The study was approved by the regional ethics committee in Gothenburg, Dnr 092-18.

## Material and methods

### The register

ENT clinics performing septoplasty in Sweden participate in the SNSR on a voluntary basis. The data in the SNSR are continuously compared with the data in the National Patient Register (NPR), where it is mandatory to report all surgical procedures performed in Sweden. The NPR includes data on diagnosis, surgical code and date of surgery and it is administered by the Swedish Board of Health and Welfare [8]. Unlike the SNSR, the NPR does not include any data on surgical outcome. The match between data in the SNSR and the NPR based on the unique personal identity number of each patient was 47.7% in 2015 and 47.8% in 2016, meaning that about half of all septoplasties performed in Sweden were reported to the SNSR.

### Inclusion criteria

**Fig. 1:**
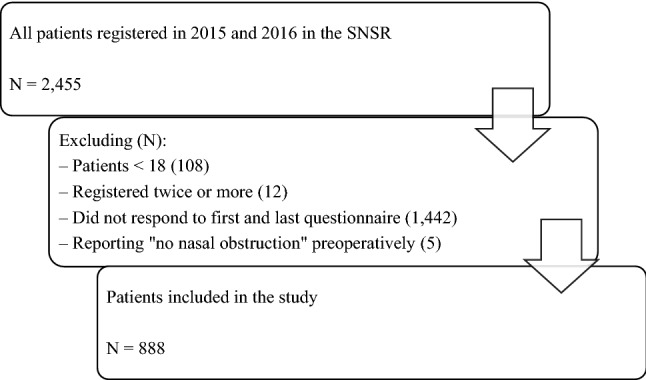
Flow chart of the study population

Patients who underwent septoplasty or septoplasty + turbinate surgery as a primary procedure due to the symptom of nasal obstruction (reporting at least mild nasal obstruction before surgery), aged 18 and above, registered in the SNSR and operated on between 2015 and 2016. Only patients who answered the questions regarding nasal obstruction both pre- and 12 months postoperatively were included (questionnaires 1 and 4) (Fig. 1).

### The questionnaires

The SNSR contains data from four questionnaires.

*Questionnaire 1* The first questionnaire has two parts. The first part is filled out by the patients where they assess their level of nasal obstruction (none, mild, moderate, severe), side of nasal obstruction, day- and/or night-time symptoms and activity impairment (none, mild, moderate, severe) due to the nasal obstruction. The question used to assess the effect of nasal obstruction on daily activities (for example, work, studies, leisure activities) and sleep has been adapted from the allergic rhinitis and its impact on asthma (ARIA) consensus document. This item was graded none, mild, moderate or severe. The second part is filled out by the ENT surgeon who diagnoses the patient and it contains data on diagnosis, side of septal deviation (right, left, both), co-morbidities, if rhinomanometry was used and planned surgical procedure.

*Questionnaire 2* The second questionnaire is filled out by the ENT surgeon performing the septoplasty and includes data on surgical technique, any nasal packaging or the use of antibiotics.

*Questionnaire 3* The third questionnaire is mailed/e-mailed to the patient one month after surgery. The patient is asked about any unplanned visits to health care due to postoperative complications (i.e., bleeding, pain, infection or other) within the first month after surgery.

*Questionnaire 4* The fourth and final questionnaire is mailed/e-mailed to the patient 12 months after surgery. The patient is again asked about the level of nasal obstruction, the impairment of nasal obstruction on daily activities and sleep and if the result was as he/she expected. Patients were also asked if they had suffered any unexpected adverse effects 12 months after the surgery.

### Statistical analyses

Patients who rated their nasal obstruction as one level better 12 months after septoplasty compared with preoperatively were defined as “improved”. Descriptive statistics are presented as percentages or mean values with standard deviations (SD). The Cochran–Armitage test was used to test for trends in ordered categorical variables. Odds ratios were calculated using logistic regression. Due to the large amount of missing data on the “Unplanned visits to health care within one month of surgery” question (32%), we used multiple imputation using the fully conditional specification method. We included the same variables as in the logistic regression model in the imputation model, with the addition of BMI, and 50 imputations were used. All the analyses were performed using SAS version 9.4M5 (SAS Inst Inc, Cary, NC, USA).

## Results

Baseline data for the study population are shown in Table 1. There was a predominance of men (71%) and the mean age was 38. The 1434 subjects who were not included in this study due to missing answers regarding pre- and/or postoperative nasal obstruction were younger (mean age 34) and included more men (77%).

**Table 1:** Study population from the Swedish National Septoplasty Register, *n* = 888, who underwent septoplasty or septoplasty + turbinate surgery in 2015–2016

*n* = 888		Missing
Septoplasty	716	
Septoplasty + turbinate surgery	172	
Mean age at surgery (years, SD)	38 (14)	0
Mean BMI (kg/m^2^, SD)	26 (4)	86
Gender, *n* (%)		0
Male	632 (71)	
Female	256 (29)	
Smoking habits, *n* (%)		23
Non-smokers	683 (79)	
Smoking daily	96 (11)	
Smoking occasionally	86 (10)	
Nasal polyps	19 (2)	82
Rhinitis	175 (22)	83
Snoring	242 (30)	84
OSAS	91 (11)	80
Nasal obstruction, *n* (%)		8
Daytime	40 (5)	
Nighttime	160 (18)	
Both	680 (77)	

**Fig. 2:**
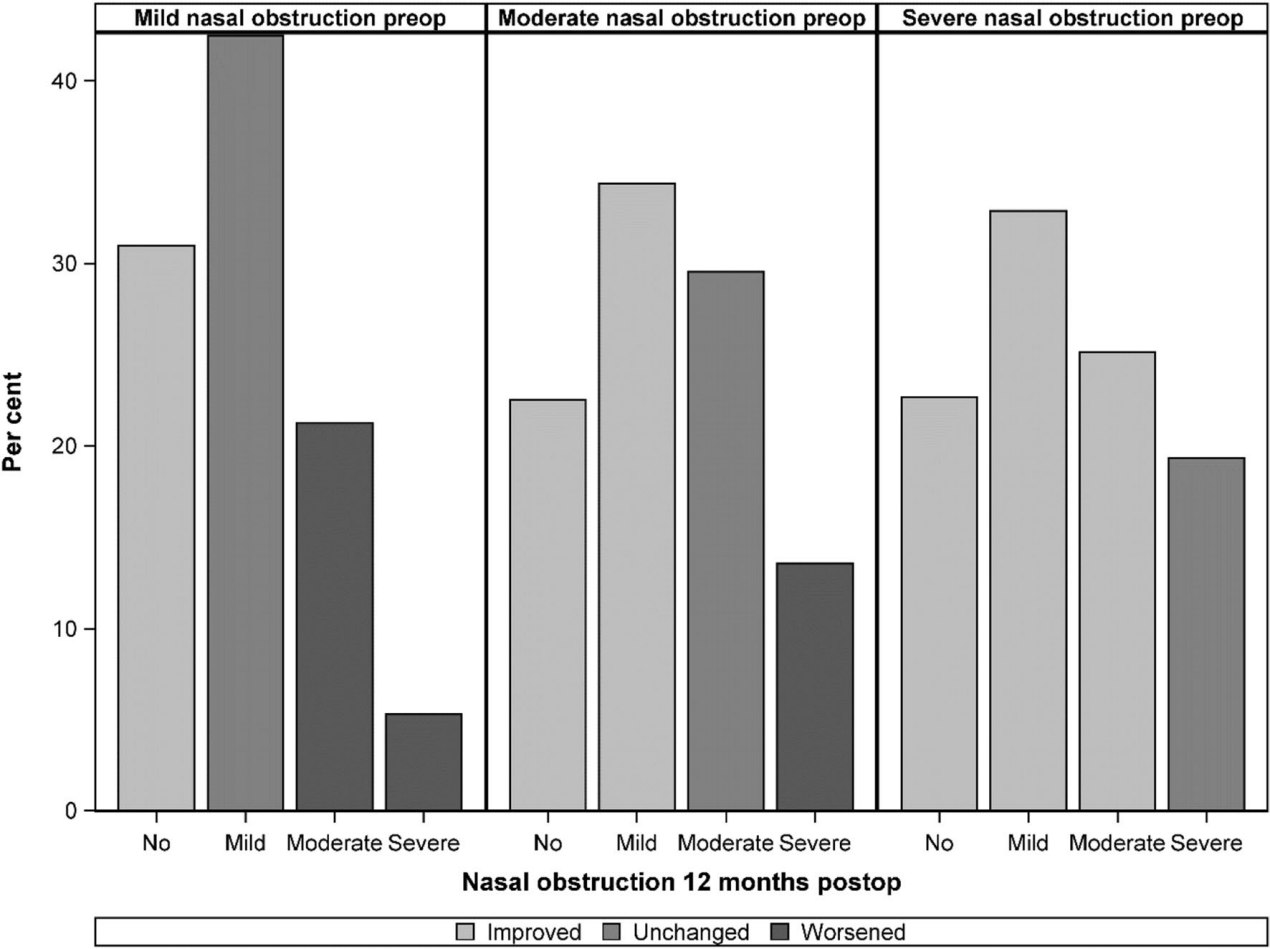
Severity of nasal obstruction 12 months postoperatively (%) in relation to self-reported nasal obstruction preoperatively (paired data) in 888 patients undergoing septoplasty. Light gray bars indicate less nasal obstruction postoperatively

A total of 63% of the patients were improved and rated their nasal obstruction as one level better after septoplasty (for example, from severe preoperatively to moderate 12 months postoperatively) (Table 2). Improvements were seen in 81% of the patients with severe nasal obstruction preoperatively and 57% of the patients with moderate nasal obstruction (Fig. 2). Activity limitation and sleep impairment due to nasal obstruction pre and postoperatively are shown in Table 3. Twelve months postoperatively, 63% of the patients with moderate to severe activity limitation and impaired sleep due to nasal obstruction preoperatively had no to mild activity limitation and sleep impairment. Activity limitation and impaired sleep were strongly related to level of nasal obstruction. The patients who improved from moderate/severe nasal obstruction before surgery to mild/none 12 months after surgery had a moderate/severe impact on their daily activities and sleep before surgery in 76% which was reduced to 7% 12 months after surgery. Agreement between the surgeon’s assessment of the side of the septal deviation (left, right or both) and the patient’s report of the side of the nasal obstruction (left, right or both) before surgery in relation to the severity of nasal obstruction 12 months after surgery is shown in Table 4.

**Table 2:** Preoperative nasal obstruction, result being as expected 12 months after surgery and any adverse events 12 months after surgery in relation to improvement 12 months after surgery (at least one level better regarding nasal obstruction, for example, from severe preoperatively to moderate postoperatively)

*n* (%)	Improved	Not improved	Total	Missing
Preoperative nasal obstruction				
Mild	35 (31)	78 (69)	113 (13)	
Moderate	235 (57)	178 (43)	413 (46)	
Severe	292 (81)	70 (19)	362 (41)	0
Result as expected 12 months postop	424/553 (77)	60/321 (19)		14
Adverse events 12 months postop	106/550 (19)	119/308 (39)		30

**Table 3:** Activity limitation due to nasal obstruction before and after surgery

	Activity limitation after surgery		
	None/mild	Moderate/severe	Total
Activity limitation before surgery			
None–mild	128 (84)	24 (16)	152 (17)
Moderate–severe	459 (63)	269 (37)	728 (83)
Total	587 (67)	293 (33)

**Table 4:** Patient-rated nasal obstruction 12 months after surgery in relation to preoperative agreement regarding side of nasal obstruction (left, right or both) between patient and ENT surgeon

	Side agree yes	Side agree no	Total
Nasal obstruction 12 months after surgery			
No	151 (73)	57 (27)	208
Mild	201 (65)	108 (35)	309
Moderate	149 (63)	87 (37)	236
Severe	78 (60)	53 (40)	131

Missing data *n* = 4

In the univariate analyses, the items of gender, smoking habits, additional turbinate surgery, allergic rhinitis, snoring, OSAS and nasal polyps were not associated with improvement after 12 months. In the regression model adjusted for age, gender, nasal obstruction preoperatively, time of day/night of symptoms, activity limitation preoperatively, the presence of allergic rhinitis and unplanned visits within one month of surgery, both higher age at surgery and no reported unplanned visits due to complications within 30 days after surgery were associated with an improvement in nasal obstruction 12 months after surgery. The most common causes of unplanned visits to health care within one month of surgery were pain (*n* = 100), bleeding (*n* = 45), infection (*n* = 56) and other causes (*n* = 73). Adverse effects reported by patients 12 months after surgery were impaired sense of smell (*n* = 80), septal perforation (*n* = 16), altered shape of the nose (*n* = 71), persistent pain (*n* = 68) and other adverse effects (*n* = 124).

## Discussion

In this study of 888 patients from the Swedish National Septoplasty Register, we found that, 12 months after septoplasty, 63% of the patients experienced an improvement in their nasal obstruction. Patients with severe nasal obstruction before surgery experienced a greater impact on their daily activities and sleep and the highest rate of improvement 12 months after surgery. The results indicate that patients with severe nasal obstruction are more likely to benefit from surgery than patients reporting mild obstruction before surgery. Higher age at surgery and agreement between doctor and patient regarding the side of the nasal obstruction before surgery was associated with a better result.

The original patient sample in this study represents around 50% of all the septoplasties carried out in Sweden (population 10 million) between 2015 and 2016. Because of missing answers regarding nasal obstruction before or after surgery, the study population was reduced. In spite of this, this cohort of 888 patients represents a significant number of septoplasties performed in one country and assessed before and 12 months after surgery. The analyses of the patients with missing data in the SNSR revealed only minor differences within the study population with regard to age, gender or nasal obstruction.

In accordance with previous studies, the majority of patients in this study undergoing septoplasty were men [1, 9, 10]. Why men are over-represented in septoplasty surgery is interesting. Septal deviation is related to trauma, which could be more prevalent in men than in women due to sports and assault. It is, however, also possible that men and women are assessed differently when seeking health care for nasal obstruction or that surgery is presented and/or accepted differently between the genders.

The main finding in this study was that patients with severe nasal obstruction before surgery benefited the most from the septoplasty. Severe nasal obstruction preoperatively represented 41% of the patients in this national cohort and, of these, 81% had improved 12 months after surgery. With moderate nasal obstruction before surgery, the improvement rate dropped to 57%, while it fell to only 31% for mild obstruction. We are not aware of any previous studies showing this relationship between the severity of symptoms and success rate in a large group of septoplasty patients. One advantage of the SNSR is that it includes patients with varying degrees of nasal obstruction, from a number of different ENT clinics where the surgery is performed by many ENT surgeons with different surgical experience. In a common surgical procedure such as septoplasty it is important to have solid predictors for the surgical outcome to improve quality of care as well as optimizing the utility of the health care resources.

Previous studies have shown that persistent nasal obstruction has a negative impact on disease-specific health-related quality of life [11–13]. In this study, we analyzed the impact of nasal obstruction on daily activities and sleep according to the ARIA (Allergic Rhinitis in Asthma Initiative) criteria. These criteria were developed for use in allergic rhinitis and may not necessarily reflect the situation in septoplasty patients. However, we found good agreement between the severity of nasal obstruction and the impact on daily activities and sleep before and after surgery. In fact, 83% of the patients had a moderate to severe limitation in daily activities and sleep prior to surgery due to their nasal obstruction and this figure fell to 33% 12 months after surgery. Since the patients in this study had a mean age of 38 years, they are of working age and the improvement in daily activities and sleep is, therefore, important both from a patient perspective and from a health-economic point of view. High costs due to nasal obstruction have previously been associated with reduced working capacity when at work, in patients with allergic rhinitis where nasal obstruction is the main symptom [14].

In accordance with our previous study from the SNSR, we found that unplanned visits to health care due to infection, bleeding, pain and other reasons within 1 month of surgery were associated with less improvement [1]. As might be expected, pain was the most common reason for unplanned visits. Infection, bleeding and pain could be associated with a defective healing process and residual nasal obstruction. This may also affect the patient’s perception of the success of the surgery and increase attention on remaining symptoms. Optimizing postoperative care to avoid unplanned visits due to infection, bleeding and pain should thus be a priority in septoplasty.

Preoperative agreement between the doctor’s assessment of the side of the deviation and the patient’s perception of the side of the nasal obstruction predicted a better outcome. It has long been arbitrary knowledge that an indication for septoplasty exists if there is agreement between the side where the symptoms are worst and the side that is blocked on inspection (including obstruction on both sides).

Higher patient age at the time of the surgery was associated with a better result 12 months after surgery, in accordance with the findings in our previous study [1]. The effect of additional turbinate surgery in septoplasty has shown conflicting results [15, 16]. In this study we were unable to find any association between additional turbinate surgery and a better result, which is also in accordance with our previous study from the SNSR [1]. With the present study design, lacking accurate data on the specific nature of the nasal obstruction it is, however, not possible to rule out the benefit of additional turbinate surgery in selected patients.

Snoring and OSAS have been associated with increased nasal airway resistance, but septoplasty has not been shown to be an effective treatment for OSAS. In this cohort of patients, we found no association between snoring and OSAS preoperatively and improvement after 12 months.

Our results support the view that septoplasty is an effective method to improve nasal obstruction due to septal deviation in well-selected patients, especially if the patient experience severe nasal obstruction before surgery. However, the fact that no improvement was seen in 69% of the patients with mild nasal obstruction before surgery and 43% of patients with moderate nasal obstruction before surgery is not satisfactory. In addition, 15% of all the patients in the study reported severe nasal obstruction 12 months after surgery. Half these patients experienced a deterioration in symptoms after septoplasty. It should also be kept in mind that a further deterioration in symptoms has been reported with a longer follow-up period after septoplasty [10].

The patients’ report of the surgical result being “as expected” was highest in the “improved” group, indicating that not only the level of nasal obstruction but also the fact that the patient experiences a change for the better after the surgery are important. Further studies are needed to determine other predictors of the outcome in septoplasty regarding surgical skill and different phenotypes of nasal obstruction involving septal deviation.

## Conclusion

This study shows that nasal obstruction improved in around 63% of the patients undergoing septoplasty and that the patients with the most severe symptoms have the best rate of improvement. These patients also experience the greatest benefit in terms of improved daily activity and sleep. Higher age at surgery and agreement between doctor and patient regarding the side of the nasal obstruction before surgery were also associated with a better result.

## References

[1] Pedersen L, Schiöler L, Holmberg K, Ahlström Emanuelsson C, Hellgren J (2018). Age and unplanned postoperative visits predict outcome after septoplasty: a national Swedish register study. Int J Otolaryngol.

[2] Jessen M, Ivarsson A, Malm L (1989). Nasal airway resistance and symptoms after functional septoplasty: comparison of findings at 9 months and 9 years. Clin Otolaryngol Allied Sci.

[3] Konstantinidis I, Triaridis S, Triaridis A, Karagiannidis K, Kontzoglou G (2005). Long term results following nasal septal surgery. Focus on patients' satisfaction. Auris Nasus Larynx.

[4] Dinis PB, Haider H (2002). Septoplasty: long-term evaluation of results. Am J Otolaryngol.

[5] Toyserkani NM, Frisch T (2012). Are too many septal deviations operated on? A retrospective patient’s satisfaction questionnaire with 11 years follow-up. Rhinology.

[6] Holmstrom M (2010). The use of objective measures in selecting patients for septal surgery. Rhinology.

[7] Haavisto LE, Sipilä JI (2013). Acoustic rhinometry, rhinomanometry and visual analogue scale before and after septal surgery: a prospective 10-year follow-up. Clin Otolaryngol.

[8] https://www.socialstyrelsen.se/register/halsodataregister/patientregistret/inenglish

[9] Karatzanis AD, Fragiadakis G, Moshandrea J, Zenk J, Iro H, Velegrakis GA (2009). Septoplasty outcome in patients with and without allergic rhinitis. Rhinology.

[10] Sundh C, Sunnergren O (2015). Long-term symptom relief after septoplasty. Eur Arch Otorhinolaryngol.

[11] Alakärppä AI, Koskenkorva TJ, Koivunen PT, Alho OP (2018). Predictive factors of a beneficial quality of life outcome in patients undergoing primary sinonasal surgery: a population-based prospective cohort study. Eur Arch Otorhinolaryngol.

[12] Alakärppä AI, Koskenkorva TJ, Koivunen PT, Alho OP (2017). Quality of life before and after sinonasal surgery: a population-based matched cohort study. Eur Arch Otorhinolaryngol.

[13] Buckland JR, Thomas S, Harries PG (2003). Can the Sino-nasal Outcome Test (SNOT-22) be used as a reliable outcome measure for successful septal surgery?. Clin Otolaryngol Allied Sci.

[14] Cardell LO, Olsson P, Andersson M, Welin KO, Svensson J, Tennvall GR, Hellgren J (2016). TOTALL: high cost of allergic rhinitis-a national Swedish population-based questionnaire study. NPJ Prim Care Respir Med.

[15] Samad I, Stevens HE, Maloney A (1992). The efficacy of nasal septal surgery. J Otolaryngol.

[16] Nunez DA, Bradley PJ (2000). A randomised clinical trial of turbinectomy for compensatory turbinate hypertrophy in patients with anterior septal deviations. Clin Otolaryngol Allied Sci.

